# Characterization of exosome-mediated propagation of systemic inflammatory responses into the Central Nervous System

**DOI:** 10.21203/rs.3.rs-4423565/v1

**Published:** 2024-06-03

**Authors:** Mahesh Chandra Kodali, Chinnu Salim, Saifudeen Ismael, Sarah Grace Lebovitz, Geng Lin, Francesca-fang Liao

**Affiliations:** Massachusetts General Hospital; Indiana University Bloomington; Tulane University; University of Tennessee College of Medicine: The University of Tennessee Health Science Center College of Medicine; University of Tennessee College of Medicine: The University of Tennessee Health Science Center College of Medicine; University of Tennessee College of Medicine: The University of Tennessee Health Science Center College of Medicine

## Abstract

The mechanisms through which systemic inflammation exerts its effect on the CNS are still not completely understood. Exosomes are small (30 to 100 nanometers) membrane-bound extracellular vesicles released by most of the mammalian cells. Exosomes play a vital role in cell-to-cell communication. This includes regulation of inflammatory responses by shuttling mRNAs, miRNAs, and cytokines both locally and systemically to the neighboring as well as distant cells to further modulate their transcriptional and/or translational states and affect the functional phenotype of those cells that have taken up these exosomes. The role of circulating blood exosomes leading to neuroinflammation during systemic inflammatory conditions was further characterized. Serum-derived exosomes from LPS-challenged mice (SDEL) were freshly isolated from the sera of the mice that were earlier treated with LPS and used to study SDEL effects on neuroinflammation. Exosomes isolated from the sera of the mice injected with saline were used as a control. *In-vitro* studies showed that the SDEL upregulate pro-inflammatory cytokine gene expression in the murine cell lines of microglia (BV-2), astrocytes (C8-D1A), and cerebral microvascular endothelial cells (bEnd.3). To further study their effects *in-vivo*, SDEL were intravenously injected into normal adult mice. Elevated mRNA expression of pro-inflammatory cytokines was observed in the brains of SDEL recipient mice. Proteomic analysis of the SDEL confirmed the increased expression of inflammatory cytokines in them. Together, these results further demonstrate and strengthen the novel role of peripheral circulating exosomes in causing neuroinflammation during systemic inflammatory conditions.

## Introduction

Propagation of inflammatory signals across the whole body via immune cells requires intercellular communication [1]. Cytokines, chemokines, and cell surface receptors play a vital role in transferring such signals between immune cells. Recent studies reveal that immune cells can also transfer inflammatory responses by secreting nano-sized lipid packages called exosomes, which carry different biomolecules including proteins, lipids and nucleic acids that could be taken up by the recipient cells [2–5]. Exosomes are membrane-bound, nanosized, homogenous population of extracellular vesicles (EVs) that are released by cells of different lineages under homeostatic as well as pathological conditions. Earlier believed to be ‘extracellular trash’, exosomes have recently initiated enormous interest after the discovery of their role in mediating intercellular communication by shuttling functional proteins, mRNA as well as microRNAs (miRNAs) to recipient cells [6].

Exosomes range in size from 30 nm to 100 nm and are encapsulated by a lipid bilayer [5, 7]. Exosomes are of endocytic origin derived from an endolysosomal pathway and are formed within multi-vesicular bodies (MVBs) by inward budding during endosome maturation [8]. Exosomes are then released by the cells into the extracellular environment upon fusion of the MVBs with the plasma membrane. Exosomal cargo sorting is governed by proteins linked with the endosomal sorting complex required for transport (ESCRT), which include ALG-2 interacting protein X (ALIX) and tumor susceptibility gene 101 (TSG101) which are the most commonly used exosomal marker proteins [9, 10]. In addition, exosomes also contain several intraluminal and transmembrane proteins including heat shock proteins (HSP70, HSP90) and tetraspanin proteins (CD9, CD63, CD81) [11].

Exosomes mediate cell to cell communication after they initially attach or fuse with a recipient cell membrane and are then internalized by the mechanism of endocytosis [12, 13]. Despite their obvious homogeneous size, exosomes mediate a broad spectrum of effects on the recipient cells indicating that different types of cells surrounded by varying physiological microenvironments, release different kinds of exosomes characterized by heterogeneity in their molecular profile [9]. Exosomal miRNAs have recently received increased attention as they can regulate the gene expression of the recipient cells in both paracrine and/or endocrine manner, suggesting exosome mediated miRNA transfer could be a novel mechanism of intercellular communication to regulate cellular function [14]. Accumulating evidence in many cells including immune cells suggests that exosomal transferred miRNA repress target genes in the recipient cells [1]. Interestingly, miRNAs can also act as signals for membrane receptor activation as they can function as ligands for toll like receptors [15]. TLR activation results in NF-kB signaling, followed by secretion of pro-inflammatory cytokines, suggesting additional role of miRNAs in inflammation, possibly by a mechanism independent of the conventional role of post-transcriptional gene regulation [16]. Recently, circular RNAs are identified as a novel class of RNAs which are generated by the mechanism of back splicing [17]. Circular RNAs function in regulating the gene as well as the miRNA expression [18]. Increasing studies indicate that these circular RNAs are enriched in exosomes derived from mammalian cells [19]. Exosomal miRNAs are also involved in the inflammatory responses and some of them like mir-155, a promoter of inflammatory responses and mir-146a, a mediator of immune suppression are secreted into exosomes during the activation of innate immune cells including dendritic cells [1]. In addition, this exosomal miRNA is transferred between dendritic cells, B-cells, myeloid cells and T-cells and further regulate the inflammatory responses to lipopolysaccharide [1].

The role of immune system activation on the brain in the context of inflammation is highly relevant for most of the neurodegenerative diseases. Yet, the mechanisms that underlie these interactions are poorly understood and needs to be elucidated in detail. As exosomes mediate intercellular immune signals in the periphery, they could possibly play a role in transmitting the same to the CNS and thus it is imperative to study the role of exosome mediated peripheral and CNS communication in detail and learn their overall contribution in causing neuroinflammation. The potential role of peripheral circulating exosomes in mediating neuroinflammation was first reported earlier [20]. In this report, we further provide additional supporting data that demonstrates the neuroinflammatory potential of the peripheral exosomes.

## Methods

### Animals

C57BL/6J mice were obtained from Jackson Laboratories, housed and bred in well-ventilated cages under standard laboratory conditions on 12:12 hour light-dark cycle with food and water ad libitum. Both male and female mice aged between eight to twelve weeks were used unless otherwise mentioned. All animal experimental procedures were conducted in accordance with the animal care standards of the National Institute of Health and were approved by the Institutional Animal Care and Use Committee (IACUC) of the University of Tennessee Health Science Center (UTHSC).

### Lipopolysaccharide (LPS) Treatment

Mice were randomized into experimental groups and received a single intraperitoneal injection of either endotoxin-free phosphate buffered saline (PBS) or LPS from *E. coli* O55:B5(Sigma-Aldrich L2880) dissolved and diluted in endotoxin-free PBS (10 mg kg^−1^ body weight [21–24]). Mice were sacrificed at specific time points post injection.

### Real Time Quantitative Polymerase Chain Reaction (RT-qPCR)

Total RNA was extracted using the Trizol method and cDNA synthesis was performed using the SuperScript^™^ IV VILO^™^ Master Mix with ezDNase^™^ Enzyme (Invitrogen, 11766050) according to the manufacturer’s protocols. All primer sequences were obtained from the primer bank [25–27]. RT-qPCR reactions were performed using 2X SsoAdvanced Universal SYBR Green Supermix (Bio-Rad, 1725271) on an Eppendorf Mastercycler Realplex2.

### Exosome Isolation, Quantification

Isolation of exosomes was done using ExoQuick serum exosome precipitation solution (EXOQ5A-1, Systems Biosciences, San Francisco, CA, USA) according to the manufacturer’s instructions. The pellets which were enriched in exosomes were dissolved in PBS. For quantifying the protein concentration of exosomes, either intact exosomes were used directly after resuspension into the PBS or were lysed in 2X radioimmunoprecipitation buffer and sonicated briefly. The lysates were then centrifuged at 12000 × g at 4°C for 20 minutes. The supernatant was then quantified by using Pierce BCA protein assay kit (23225, Thermo Fisher Scientific, Waltham, MA, USA).

### Transfusion of Serum-Derived Exosomes from Donor to Recipient Mice

Whole blood (700–800 μl) from mice was collected by cardiac puncture. Blood was centrifuged at 2,000 × *g* for 10 min after sitting undisturbed at room temperature for 30 minutes to separate the serum. Purified exosomes from the sera were then resuspended in 200 μl of sterilized 1X PBS and passed through a 0.22 μm filter before intravenous (IV) tail vein injection to the recipient mice.

### Cell culture

Immortalized microglial cells (BV-2) (Accegen, ABC-TC212S), astrocytes (C8-D1A) (ATCC, CRL-2541) and mouse endothelial cells (bEnd.3) (ATCC, CRL-2299), were cultured in Dulbecco’s modified Eagle’s medium (DMEM; Gibco, NY, USA) with 10% fetal bovine serum (FBS; Gibco, NY, USA) in a 5% CO incubator at 37°C. Cells were treated for 24 hours with 0.1 mg/ml of exosomes derived from the serum of the donor mice injected with either LPS or PBS for 24 hours.

### Cytokine analysis

Exosome homogenates were prepared in RIPA buffer (Sigma, St. Louis, MO) was centrifuged (21,000 g x 30 min) and supernatants removed for analysis. Supernatant cytokines levels were determined using the MesoScale Discovery (MSD, Rockville, MD) 96-well Mouse Pro-Inflammatory V-PLEX Assay (MSD, K15048D-1) according to the manufacturer’s instructions. Briefly, samples are incubated in wells containing an array of cytokine capture antibodies directed against Interleukins IL-6, KC/GRO, TNF-α, IL-12p70, IFN-γ, IL-1β, IL-2, IL-4, IL-5 and IL-10. Bound cytokines are detected immunochemically, and the signal was read using an MSD Sector Imager 6000.

### Statistics

All statistical analyses were conducted using GraphPad Prism v. 8 (GraphPad Software, San Diego, CA). Data are presented as mean ± standard error of the mean (SEM). The sample size for each experiment, and the statistical testing methods are reported in the figure legends.

## Results

### Systemic inflammatory conditions increase exosome secretion as well as total exosomal protein content

**Figure 1.**
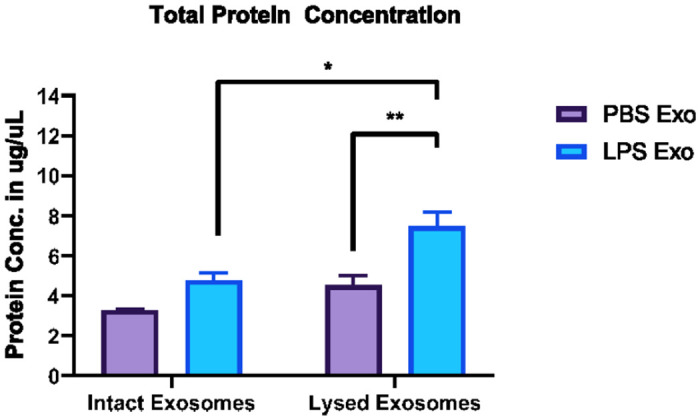
Total protein concentration of intact and lysed exosomes isolated from the sera of the mice injected with either LPS or PBS for 24 hours, n = 3-4 per group Two -way anova with Sidak’s multiple comparisons test. Data represent mean ± SEM; *p<0.05, **p<0.01 as compared to the PBS group)

To know if the exosome secretion is altered during systemic inflammation, we determined the total protein content of the exosomes isolated from the sera of the mice which were earlier injected with LPS or PBS. Our earlier results suggested that exosome secretion is indeed increased after LPS injection [20], suggesting the effect of systemic inflammation on exosome secretion. Furthermore, by quantifying the total protein amounts of the exosomes isolated from the serum of the mice after LPS or PBS injection, we concomittantly found the increase of total protein content of the exosomes (Fig. 1).

### Serum-derived exosomes purified from LPS-challenged mice activate microglia, astrocytes, and cerebral microvascular endothelial cells

**Figure 2.**
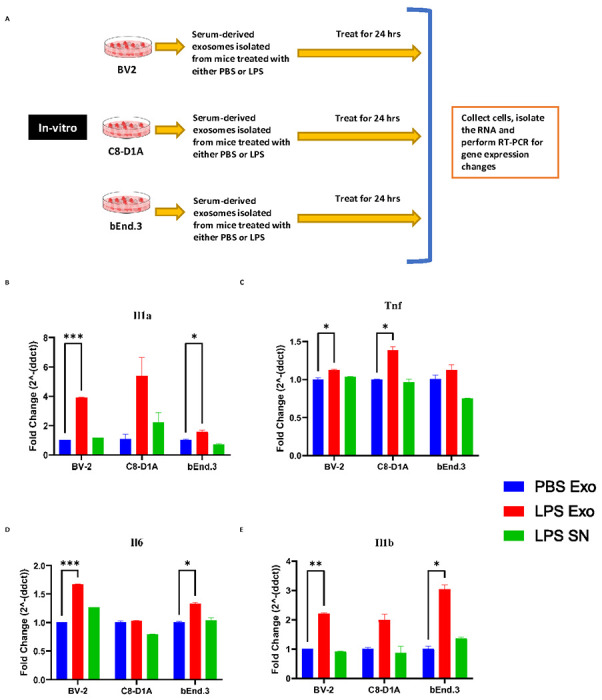
(A) Scheme depicting the in vitro experimental design for investigating the effects of serum derived exosomes. (B-E) mRNA fold change levels of various proinflammatory genes in the cell lines BV-2 (microglia), C8-D1A (astrocytes) and bEnd.3 (brain endothelial cells) treated for 24 hours with 0.1 mg/ml of exosomes derived from the serum of the donor mice that were injected with PBS (PBS Exo), LPS (LPS Exo) or LPS derived exosome supernatant (LPS SN) for 24 hours, (B) *Il1a* (C) *Tnf*, (D) *Il6*, (E) *Il1b* (n= 3-4 male mice in each group, Data represent mean ± SEM; Two-way anova with Dunnett post-hoc test, *p<0.05, **p<0.01, ***p<0.001)

To investigate the neuroinflammatory potential of peripheral circulating exosomes during systemic inflammation, serum-derived exosomes purified after LPS challenge (SDEL) were used. Exosomes were isolated from serum of the mice which were injected with either PBS or LPS 24 hours earlier. Cultured BV2, C8-D1A and Bend.3 cells were then incubated with the isolated exosomes, as well as the supernatant from the exosomes precipitated from the mice treated with LPS (Fig. 2. A). Our results revealed that the exosomes derived from the mice injected with LPS, but not PBS increased the expression of proinflammatory cytokine genes in the treated cells (Fig. 2. B-E). Additionally, the supernatant from the exosome precipitation of sera derived from the mice injected with LPS, did not induce an inflammatory response in the cells.

### Serum-derived exosomes purified from LPS-challenged mice increase proinflammatory gene expression in the brain

**Figure 3.**
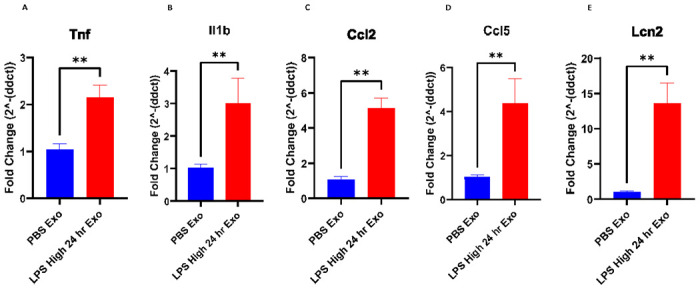
(A-E) mRNA fold change levels of various proinflammatory genes in the brains of the mice that were intravenously injected with 1 mg of exosomes derived from the serum of the donor mice injected with LPS or PBS for 24 hours, (A) *Tnf* (B) *Il1b*, (C) *Ccl2*, (D) *Ccl5 (E) Lcn2,* (n= 3-6 male mice in each group, Data represent mean ± SEM; Mann-Whitney t test, *p<0.05, **p<0.01, ***p<0.001)

Exosomes isolated from sera of the mice that were injected with either PBS or LPS 24 hours earlier were injected to recipient mice via tail vein. Brains were then isolated from the recipient mice 24 hours after the tail vein injection and analyzed for the expression of proinflammatory genes. Proinflammatory cytokines *Il1b, Tnf,* chemokine genes *Ccl2, Ccl5* and pan reactive gene *Lcn2* were found to be upregulated in the brains of the mice injected with the exosomes isolated from the sera of LPS injected mice but not PBS injected mice (Fig. 3. A-E).

### Serum-derived exosomes purified from LPS-challenged mice has elevated levels of proinflammatory cytokines

**Figure 4.**
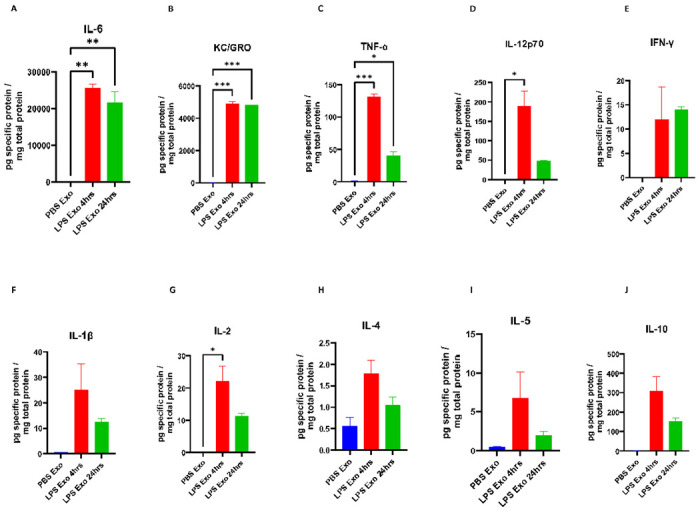
(A-J) Cytokine levels in the exosomes derived from the serum of the mice that were earlier treated with either PBS or LPS for 4 and 24 hours. (A) IL-6, (B) KC/GRO, (C) TNF-α, (D) IL-12p70, (E) IFN-γ, (F) IL-1β, (G) IL-2, (H) IL-4, (I) IL-5 and (J) IL-10 (n= 3-4 male mice in each group, Data represent mean ± SEM; One-way anova, *p<0.05, **p<0.01, ***p<0.001)

As we observed increased protein content in the exosomes during systemic inflammatory conditions (Fig. 1), we thus wanted to know if there is any change in the proinflammatory cytokine levels in these exosomes. We thus measured the levels of proinflammatory cytokines in the exosomes isolated from sera of the mice that were earlier injected with either PBS or LPS for 4 and 24 hours. Our results showed the elevated levels of proinflammatory cytokines IL-6, KC/GRO, TNF-α, IL-12p70, IFN-γ, IL-1β, IL-2, IL-4, IL-5, and IL-10 in the exosomes isolated from the mice treated with LPS but not PBS (Fig. 4. A-J). This suggests that the exosomes during inflammatory conditions have increased amounts of proinflammatory cytokines, further confirming our earlier hypothesis that these are the carriers of protein cargo during systemic inflammation.

## Discussion

This study investigated a novel mechanism of peripheral contribution to neuroinflammation mediated by circulating exosomes. Initially the microglial and astrocytic activation in the brain was demonstrated by using the peripheral LPS induced neuroinflammation model which is consistent with many other reports [28]. Next, the role of serum-derived exosomes was investigated to know if they can cause neuroinflammation during acute systemic inflammation induced by a single intraperitoneal LPS injection.

The finding of increased exosome protein concentration as determined by intact as well as lysed protein quantification of exosomal fractions after LPS injection suggests that there is an increase in the number of secreted exosomes and also points towards the alteration in the exosomal content and cargo during systemic inflammatory conditions, indicating a possible role of exosomes in mediating intercellular communication during inflammatory conditions.

Exosomes derived from the sera of mice challenged with LPS caused significant upregulation of proinflammatory genes *Il1b, Tnf, Il6* and *1l1a* in cultured BV2, C8-D1A and Bend.3 cells, demonstrating the inflammatory potential of the exosomes during systemic inflammation. More interestingly, the supernatant obtained after the precipitation of exosomes was not able to induce such effect, suggesting that the exosomes mediate such functional effects in the recipient cells during inflammatory conditions and communicate with the distant cells. Furthermore, these exosomes when injected via tail vein led to an increase in the proinflammatory cytokine genes *Tnf Il1b,* chemokine genes *Ccl2, Ccl5,* and pan reactive gene *Lcn2*. This suggests that the exosomes might be able to carry proinflammatory cargo from the periphery into the CNS and further induce neuroinflammation. However, this could also be due to the peripheral immune responses to the injected exosomes, which can also further cause neuroinflammation. Additionally, the exosomes derived from the sera of the mice injected with LPS but not PBS had elevated levels of proinflammatory cytokines IL-6, KC/GRO, TNF-α, IL-12p70, IFN-γ, IL-1β, IL-2, IL-4, IL-5 and IL-10.

Taken together these results suggest that peripheral circulating exosomes during systemic inflammatory conditions can undergo alteration in their content and are further capable of reaching the CNS to induce the upregulation of proinflammatory cytokines, and also activate microglia, astrocytes and cerebral endothelial cells and finally cause neuroinflammation. These observations suggest that exosomes may serve as an additional way in mediating the activation of neuroinflammatory processes during systemic inflammatory conditions and help to understand the mechanism of communication between the brain’s immune system and the peripheral immune system.

However, it is important to know the role of choroid-plexus epithelium (CP) and blood brain barrier endothelium in mediating the transport of SDEL from the periphery and the CNS. Recently it is shown that epithelial cells of the CP sense and relay the peripheral inflammatory status to the CNS by releasing the extracellular vesicles into the CSF, which transfer this pro-inflammatory message to recipient brain cells [29]. Nevertheless, it is still unclear if the peripheral inflammatory exosomes have any preference in mediating this effect on the CP epithelial cells compared to BBB endothelial cells, and if the exosomes released by the peripheral immune cells travel into the CNS via the CP and/or BBB.

Further studies are required to primarily understand the precise mechanism by which exosome mediated neuroinflammation occurs, to know which cells in the CNS are the recipients of peripheral exosomes during systemic inflammatory conditions. It is also worthwhile to know if exosomal biogenesis associated with miRNA packaging by the peripheral immune effector cells contributes to the neuroinflammatory signaling cascade in the CNS.

## Data Availability

All data supporting the findings of this study are available within the paper.
